# The 14th Ile residue is essential for Leptin function in regulating energy homeostasis in rat

**DOI:** 10.1038/srep28508

**Published:** 2016-07-05

**Authors:** Shuyang Xu, Xianmin Zhu, Hong Li, Youtian Hu, Jinping Zhou, Di He, Yun Feng, Lina Lu, Guizhen Du, Youjin Hu, Tiancheng Liu, Zhen Wang, Guohui Ding, Jiayu Chen, Shaorong Gao, Fang Wu, Zhigang Xue, Yixue Li, Guoping Fan

**Affiliations:** 1 Tongji University, School of Life Sciences and Technology, 1239 Siping Road, Shanghai 200092, China; 2 Key Laboratory of Systems Biology, Institute of Biochemistry and Cell Biology, Shanghai Institutes for Biological Sciences, Chinese Academy of Sciences, Shanghai 200031, China; 3 Shanghai Center for Systems Biomedicine, Shanghai Jiao Tong University, 800 Dong Chuan Road, Shanghai 200240, China; 4 Translational Center for Stem Cell Research, Tongji Hospital, Department of Regenerative Medicine, Tongji University School of Medicine, Shanghai 200065, China; 5 Department of Human Genetics, David Geffen School of Medicine, University of California Los Angeles, CA 90095 USA

## Abstract

LEPTIN (LEP) is a circulating hormone released primarily from white adipocytes and is crucial for regulating satiety and energy homeostasis in humans and animals. Using the CRISPR technology, we created a set of *Lep* mutant rats that carry either null mutations or a deletion of the 14^th^ Ile (LEP^∆I14^) in the mature LEP protein. We examined the potential off-target sites (OTS) by whole-genome high-throughput sequencing and/or Sanger-sequencing analysis and found no OTS in mutant rats. Mature LEP^∆I14^ is incessantly produced and released to blood at a much elevated level due to the feedback loop. Structure modeling of binding conformation between mutant LEP^∆I14^ and LEPTIN receptor (LEPR) suggests that the conformation of LEP^∆I14^ impairs its binding with LEPR, consistent with its inability to activate STAT3-binding element in the luciferase reporter assay. Phenotypic study demonstrated that *Lep*^∆*I14*^ rats recapitulate phenotypes of *Lep*-null mutant rats including obesity, hyperinsulinemia, hepatic steatosis, nephropathy, and infertility. Compared to the existing *ob/ob* mouse models, this *Lep*^∆*I14/*∆*I14*^ rat strain provides a robust tool for further dissecting the roles of LEP in the diabetes related kidney disease and reproduction problem, beyond its well established function in regulating energy homeostasis.

LEPTIN (LEP), a secreted peptide by white adipocyte tissues (WAT), is one of the most widely studied adipokines that regulate mammalian body weight and maintain energy balance. LEP and its receptor (LEPR), through their downstream signaling pathways, precisely execute a variety of important functions such as energy homeostasis, glucose and lipid metabolism, neuroendocrine, immune systems, reproduction, etc^1^,^2^. Briefly, the long isoform of LEPR (LEPRb), mainly localized in hypothalamus, is the most prevalent LEPR that mediates LEP function among the five known human isoforms^3^,^4^. The binding of LEP to LEPR can initiate many downstream signaling pathways^2^, including Janus kinase 2 (JAK2)-signal transducer and activator of transcription 3 (STAT3), insulin receptor substrate (IRS)-phosphatidylinositol 3-kinase (PI3K), SH2-containing protein tyrosine phosphatase 2 (SHP2)-mitogen-activated protein kinase (MAPK), and 5′ adenosine monophosphate-activated protein kinase (AMPK)/acetyl-CoA carboxylase (ACC), which are negatively regulated by suppressor of cytokine signaling 3 (SOCS3) and protein tyrosine phosphatase 1B (PTP1B).

In human, mutations of LEP lead to symptoms such as hyperphagia, obesity, hypothyroidism, hyperinsulinemia, hyperlipidemia and hypogonadism. The first congenital LEP deficiency case was reported in 1997^5^. The Pakistani cousins had homozygous deletion of guanosine at Codon 133 (∆133G) on LEP which resulted in premature stop of translation via frame shift. Interestingly, another Pakistani child was reported to have the same ∆133G mutation inherited from their heterozygous parents originally from the same geometrical area, although the different families were not genetically related for at least 4 generations^6^. Similar symptoms were reported in different families with the homozygous missense mutations on LEP, i.e., R105W^7^, N103K^8^, L72S^9^, Q55X^10^, and D100Y^11^. In all the cases, the clinical phenotypes of congenital LEP deficiency can be corrected by the LEP replacement treatment with human recombinant LEP^6^,^11^,^12^.

The animal models offer a great tool to study the pathological mechanisms of congenital LEP- and LEPR- deficiency. Classic *Lep*- and *Lepr*- mutant mice including *ob/ob*^13^, *db/db*^14^, *s/s*^15^ are well studied in the past six decades. Comparing to mouse, rat is a more suitable animal model for studying many diseases such as blood diseases, endocrine disorders, and metabolic disorders because of its physiological similarity to human^16^. For a long time, few LEPR deficiency rats were available from spontaneous mutation such as Zucker (*fa/fa*) rats^17^, Zucker Diabetic Fatty (*ZDF*) rats^18^, Koletsky rats^19^,^20^, spontaneously hypertensive/NIH corpulent (*SHR/N-cp*) rats^21^ and *JCR:LA-cp* rats^22^, which possess similar phenotypes to those of *db/db* mice. Recent advancement in genome editing techniques such as zinc finger nucleases (ZFNs), transcription activator-like effector nuclease (TALEN) and clustered regularly interspaced short palindromic repeats (CRISPR) made it feasible to create germline mutations in a variety of mammalian species including rats^23^. In 2012, the first strain of *Lep* knockout rats was created by introducing 151-bp deletion of the first exon via ZFN^24^, which are useful to study rat LEP in regulating glucose homeostasis and insulin resistance^25^. Compared to ZFN and TALEN, CRISPR is more cost-effective and efficient to make mutant rats. In this study, we successfully create rat models for LEP- and LEPR- deficiency by one-step zygotic injection of Cas9 mRNA and sgRNAs targeting rat *Lep* and *Lepr* exons. We found that *Lep* mutant rats carrying homozygous deletions of 3 nucleotides (ATC) encoding isoleucine at position 14 (I14) in the mature LEP protein exhibited similar mutant phenotypes to LEP- and LEPR- null rats. Our molecular analyses suggest that I14 is of great importance for the interaction between LEP and LEPR and the down streaming JAK2-STAT3 pathways.

## Results

### Generation of *Lep* mutant rats by CRISPR/Cas9

**Figure 1:**
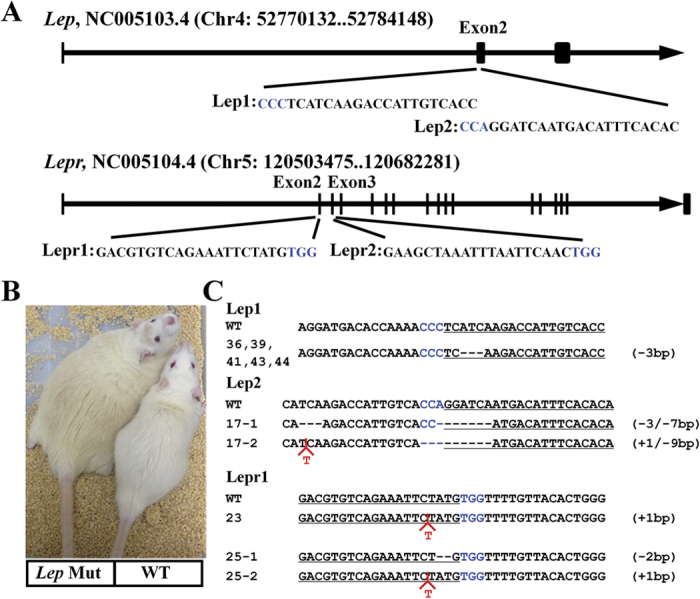
Generation of *Lep* and *Lepr* mutant rats with the CRISPR/Cas9 system. (**A**) DNA sequences of the targeting sites at *Lep* and *Lepr* loci. The sgRNA targeting sequence is shown in black, and the PAM sequence is shown in blue. (**B**) A male Lep Mutant rat (# 11) showed obese phenotype at age of wk-12 compared to a male WT rat. (**C**) Representative sequencing results of the mutant alleles in the founders. The gRNA-targeting sequences are underlined and the PAM is highlighted in blue. The deletions are indicated as – whereas the insertions are in red. The number of insertions (+) or deletions (−) are shown to the right of each allele.

We created the transgenic rats by CRISPR/Cas9 gene editing tool following a routine procedure^26^. *Lep* gene has 3 exons encoding a 167-aa protein. The mature LEP is 146 aa after the cleavage of a 21 aa N-terminal signal peptide. To generate *Lep* mutant rat models, we selected two specific CRISPR targeting sequences in the 2^nd^ exon (Fig. 1A), aiming to introduce mutations via non-homologous end joining (NHEJ) at the N-terminus of the mature LEP. Similarly, we designed CRISPR targeting sequences in the 2^nd^ and 3^rd^ exon respectively of *Lepr* gene (Fig. 1A), excluding the sequences encoding the N-terminal signal peptide. After cloning these CRISPR targeting sequences into pX330 vector, we performed the T7EI assay to test our CRISPR/Cas9 system *in vitro*. We found that each targeting sequence results in mutations so that the mismatched PCR products were cleaved by T7EI as expected (Supplementary Materials, Fig. S1). Then we did *in-vitro* transcription of both gRNAs and Cas9 mRNA, and injected each one-cell embryo with a concentration at 50 ng/μL and 100 ng/μL respectively. After oviduct implantation, we obtained viable pups of Sprague Dawley (SD) rats as summarized in Table 1. With Lep1 and Lep2 sgRNAs, we obtained 12 and 5 pups delivered respectively. In contrast, we got very few *Lepr* mutant rats from both Lepr constructs, i.e., only two pups for Lepr1 sgRNA and none for Lepr2 sgRNA, although 40 embryos were transferred for each construct. The morbidly obese phenotype was soon recognized in both *Lep*- and *Lepr*- mutant rats as early as week 3 (Fig. 1B). As expected, the genotyping results confirmed that all the obese rats carry specific mutations near each gRNA targeting site of *Lep* and *Lepr* respectively (Fig. 1C). We did not observe any heterozygote or mosaicism in the mutant rats (Table 1), most likely due to the high concentration of gRNAs and Cas9 mRNA. Besides the compound heterozygous rats, we also found five *Lep* mutant rats with the same homozygous mutation that is a deletion of three nucleotides (ATC) encoding I14 in the mature LEP protein. By reducing the concentration of both gRNAs and Cas9 mRNA at 25 ng/μL, we obtained mutant animals with mosaicism (Table 1), making germline transmission possible. Interestingly, the same genotype of ATC deletion reoccurred after the second round of zygote microinjection, which offered us an opportunity to study the *Lep* mutant phenotype caused by missing one single amino acid.

### Targeting Specificity of the CRISPR/Cas9 System

Off-target effect (OTE) has been discovered since the emergence of CRISPR technology^27^. The targeting specificity is largely determined by several factors, such as the concentration of Cas9/gRNA complexes, the number, position and distribution of mismatches between gRNA and target DNA. We asked the question whether OTE existed in the founders and influenced the phenotypes we observed. This led us to evaluate OTE in two founders with the most severe obese phenotypes, i.e., the *Lep* mutant rat #17 and the *Lepr* mutant rat #23, by whole-genome sequencing (WGS). The analysis pipeline was shown in Supplementary Materials, Fig. S2.

**Figure 2:**
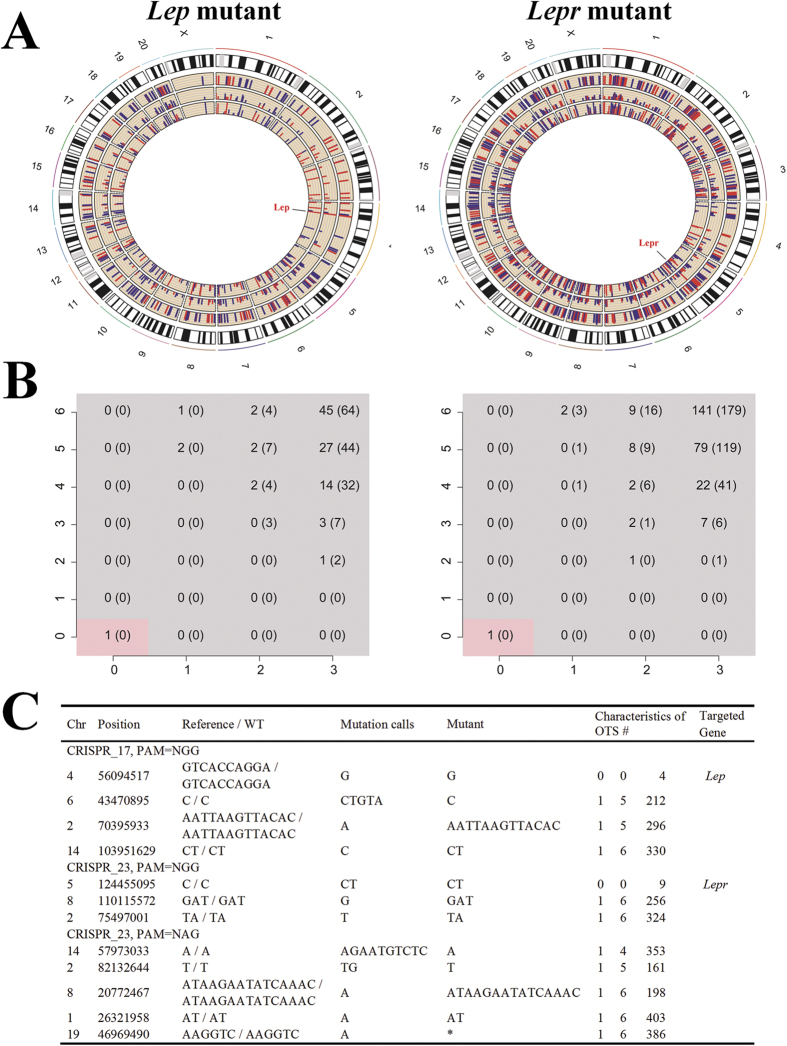
Verification of Off-target effect in the *Lep*- and *Lepr*- mutant founders generated by CRISPR/Cas9 system. (**A**) Circos plot showing the characteristics of potential OTS in the *Lep* mutated and *Lepr* mutated rats. The location of target gene is labeled by red. sgRNA targeting loci were predicted by CasOT using two PAM sequences NGG (red) or NAG (blue). Tier 1, 2 present the percentage of matched nucleotides in sgRNA seed and non-seed region, with higher value indicating more similar. Tier 3 shows the distance from OTS to sgRNA targeting loci, with higher value indicating more close. (**B**) Number of OTS under the given similarity between sgRNA and predicted target loci. PAM is NGG or NAG (number in bracket). X and Y axis display the number of mismatched nucleotides in sgRNA seed and non-seed region. (**C**) Top ranked OTS were verified by Sanger sequencing, which confirmed that there was no OTS in the tested rats. (# characteristics of OTS: number of mismatches in seed and non-seed region, distance from mutation sites to predicted target loci. *: cannot be Sanger sequenced).

We obtained high quality reads with the percentage of mapped reads ranging from 93% to 96%, which have 35.5X, 38.6X and 38.1X depths for #17, #23 and wild type (WT) rats, respectively. We identified average 4.6 million SNPs and 1.1 million indels in each rat genome by the GATK pipeline. Since indel is the most possible mutation type caused by CRISPR/Cas9, we filtered the indels and selected those as potential OTS that fulfill the following three requirements: 1) The private indels are only found in the mutant rats, but not in WT rat, 28 published rat genomes, 2 sequenced rats in our previous study (unpublished data) and dbSNP database; 2) They are not in the repeat regions or in regions with GC-content bias; 3) They locate within 500 bp of computationally predicted Cas9-gRNA targeting loci. As illustrated in Fig. 2A, we ranked these OTS by their similarity to sgRNA sequence (number of mismatches in seed region, number of mismatches in non-seed region), and their distance to predicted targeting loci. For both *Lep* (#17) and *Lepr* (#23) mutant rats, as expected, the whole-genome sequencing retrieved the mutation sites that exactly match the gRNA targeting sequence. When allowing more mismatches between the predicted targeting loci and sgRNA sequence, we found that the number of potential OTS increases (Fig. 2B). To verify the potential OTS, we amplified ~600 bp DNA fragments flanking the top ranked mutations (<2 mismatches in sgRNA seed region), and subcloned them into sequencing vector followed by Sanger sequencing. Since some DNA sequences of WT SD rats are different from those of UCSC rn5 most likely due to the strain variation (data not shown), we referred to the potential OTS regions of WT SD rats as control. After the integrated analysis, we concluded that there is no OTS in the tested *Lep* (#17) and *Lepr* (#23) mutant rats (Fig. 2C). Neither did we found any OTS in the other mutant rats after verification by the conventional method as previously described^26^ (data not shown).

### Phenotypic assessment of the *Lep* ^∆*I14/*∆*I14*^ rats

**Figure 3:**
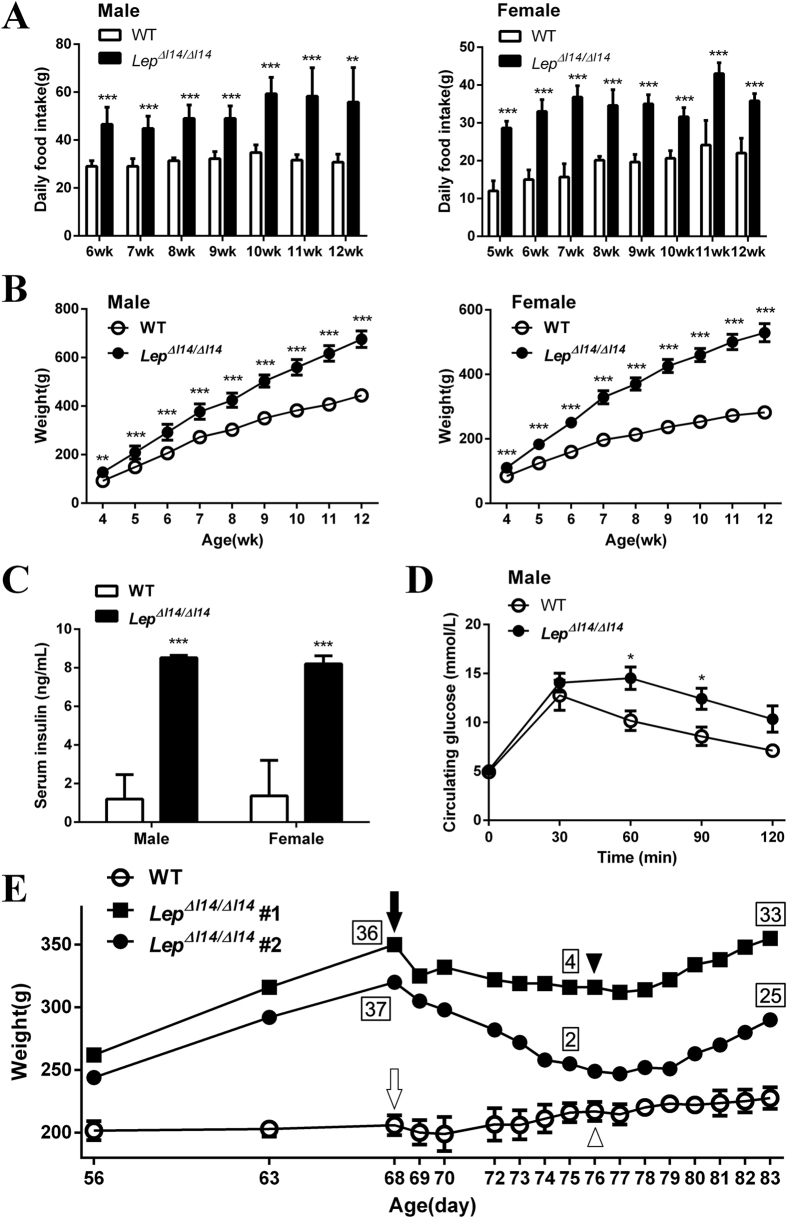
LEP-deficiency induced obesity, glucose intolerance, and hyperinsulinemia in Lep rats. (**A**) Daily food intake (Means ± SD) for WT (n = 5) and *Lep*^∆*I14/*∆*I14*^ (n = 6) measured over 7–8 weeks. *P < 0.05 vs. controls. **P < 0.01 vs. controls. ***P < 0.001 vs. controls. (**B**) Body weight was measured over 9 weeks for WT (n = 5) and *Lep*^∆*I14/*∆*I14*^ males (n = 6) and WT (n = 6) and *Lep*^∆*I14/*∆*I14*^ females (n = 6). *P < 0.01 vs. controls. **P < 0.01 vs. controls. ***P < 0.001 vs. controls. (**C**) Serum INSULIN level in 8-wk-old male WT (n = 4) and *Lep*^∆*I14/*∆*I14*^ rats (n = 5), and female WT (n = 4) and *Lep*^∆*I14/*∆*I14*^ rats (n = 6). ***P < 0.001 vs. controls. (**D**) Male WT (n = 5) and *Lep*^∆*I14/*∆*I14*^ rats (n = 6) at age of wk-16 were ip injected with D-glucose, and serum glucose levels were determined at 0, 30, 60, 90, and 120 min after administration. *P < 0.05 vs. controls. (**E**) Hyperphagia and obesity were rescued by subcutaneous infusion of recombinant LEP. Two individual female *Lep*^∆*I14/*∆*I14*^ rats were treated with rat recombinant Lep via subcutaneous implantation of an Alzet osmotic minipump (200 μg/kg/day) for 7 days before switching LEP to saline, while the WT female controls (n = 3) were implanted with a saline-filled minipump. For *Lep*^∆*I14/*∆*I14*^ rats, solid arrow indicates the start of LEP treatment; solid arrowhead indicates the switch of LEP to saline. For WT controls, open arrow indicates the start of minipump implantation; open arrowhead indicates withdrawal of minipump. Numbers near each body-weight point are the daily food intake (g) measured on the same day.

**Figure 4:**
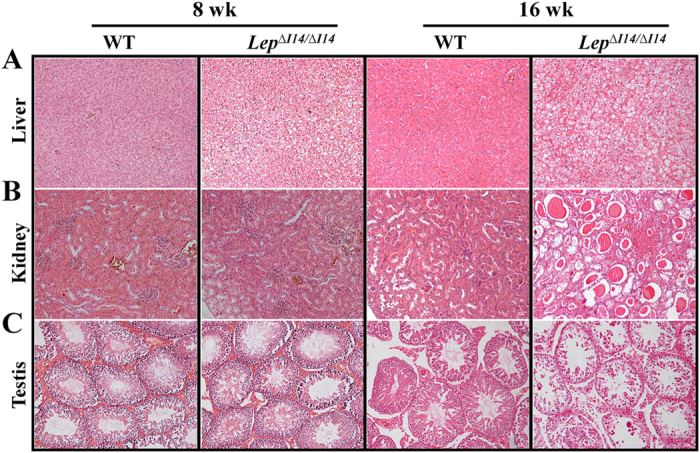
Morphological examination of liver, kidney and testis revealed the phenotypes of hepatic steatosis, nephropathy, and infertility in *Lep*^∆*I14/*∆*I14*^ rats. (**A**) *Lep*^∆*I14/*∆*I14*^ rats have many hepatocyte vacuoles in liver, a typical phenomenon of non-alcoholic fatty liver disease (NAFLD). The phenotype is progressed from week 8. (**B**) The kidney of *Lep*^∆*I14/*∆*I14*^ rats shows glomeruli hypertrophy, dilation of tubules, and many protein casts within tubules. The phenotype was observed at week 16. (**C**) The testis of *Lep*^∆*I14/*∆*I14*^ rats has hollow seminiferous tubules losing many germ cells in the intermediate and adluminal layers, which is more severe at week 16. Sections were stained with HE and photographed at 100× magnification.

As we found no OTS in the exampled animals as stated above, the obese phenotype of the mutant rats (Fig. 1B) must be resulted from LEP- and LEPR- deficiency. We next focused our study on the *Lep* mutant rats after establishing a colony of mutant *Lep* rats with deletion of three nucleotides encoding I14, namely *Lep*^∆*I14/*∆*I14*^. We measured the phenotype-related parameters of the founders in two sexes separately (Supplementary Materials, Fig. S3, Tables S1 and S2) because of the sexual dimorphism of the circulating LEP level in rats^28^. By monitoring the daily food and water intake during a 7~8-week window, we found that both male and female *Lep*^∆*I14/*∆*I14*^ rats consumed more food compared to the WT controls (Fig. 3A). The *Lep*^∆*I14/*∆*I14*^ rats started to become significantly heavier than the WT controls at week 4 and remained heavier until week 12 (Fig. 3B) and throughout their lives (data not shown). As shown in Table 2, we found that all the serum parameters, i.e. triglyceride, cholesterol, high-density lipoprotein, and low-density lipoprotein are significantly higher in both male and female *Lep*^∆*I14/*∆*I14*^ rats compared to the WT controls. As expected, we observed hyperinsulinemia phenotype in both male and female *Lep* mutant rats. The serum INSULIN level of the male *Lep*^∆*I14/*∆*I14*^ rats was significantly higher than the WT controls (8.52 ± 0.12 ng/ml vs. 1.20 ± 1.26 ng/ml in WT, Mean ± SD), so was that of the females (8.21 ± 0.41 ng/ml vs. 1.36 ± 1.84 ng/ml in WT, Mean ± SD) (Fig. 3C). We also performed glucose tolerance experiment in the *Lep*^∆*I14/*∆*I14*^ rats at week 16. After injecting D-glucose intraperitoneally, we collected blood samples at 30, 60, 90, and 120 min and read serum glucose concentration. As shown in Fig. 3D, we observed that serum glucose level in the male mutant rats was significantly higher than that in the WT controls at 60 min (14.52 ± 2.80 vs. 10.18 ± 2.24 mmol/L in WT, Mean ± SD) and at 90 min (12.42 ± 2.65 vs. 8.58 ± 2.10 mmol/L in WT, Mean ± SD). We then asked whether treatment of exogenous LEP can rescue the obesity phenotype. We subcutaneous implanted two female *Lep*^∆*I14/*∆*I14*^ rats with an Alzet osmotic minipump which can continuously release rat recombinant LEP at a dosage of 200 μg/kg/day for 7 days. As shown in Fig. 3E, treatment with saline alone did not result in significant fluctuation in body weight of the WT controls. But both *Lep*^∆*I14/*∆*I14*^ rats lost substantial weight during the 7-day period of LEP treatment (From average 335 g on day 1 to average 285.5 g on day 7, Fig. 3E), parallel with reduced daily food-intake (From average 36.5 g on day 1 to average 3 g on day 7, Fig. 3E), consistent with the treatment effect in *ob/ob* mice^29^,^30^ and patients with congenital LEP deficiency^6^,^11^,^12^. When LEP was withdrawn and replaced with saline, we observed that both body-weight and daily food-intake of *Lep*^∆*I14/*∆*I14*^ rats were dramatically increased again. Interestingly, morphological examination of organs in *Lep*^∆*I14/*∆*I14*^ rats revealed the abnormalities in the organs such as liver, kidney and testis, however, the onset of kidney phenotype is late compared to that of liver and testis phenotypes (Fig. 4).

### Essential role of Ile14 for LEP-LEPR binding

**Figure 5:**
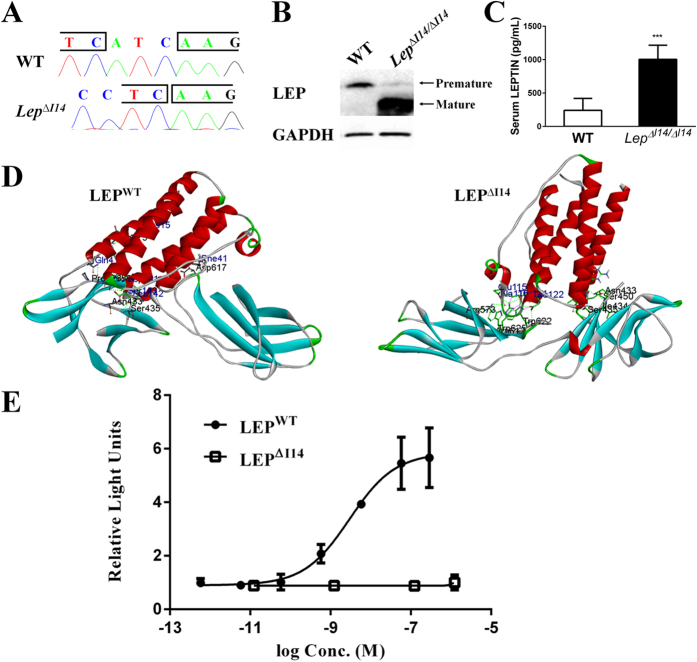
I14 in mature LEP is essential for LEP-LEPR interaction and the downstream STAT3 pathway. (**A**) Sanger-sequencing of the RT-PCR products showed that the *Lep* mRNA of *Lep*^∆*I14/*∆*I14*^ rat had a deletion of 3 nucleotides ATC encoding an Ile residue. (**B**) Western blot showed that the mature LEP^∆I14^ protein is stably expressed in the WAT of *Lep*^∆*I14/*∆*I14*^ rats. Shown is one of three independent experiments. (**C**) ELISA showed that serum LEP^∆I14^ in male *Lep*^∆*I14/*∆*I14*^ rats (n = 5) is significantly increased compared to that of serum LEP^WT^ in the male WT controls (n = 5). (**D**) Computer assimilation of LEP-LEPR interaction using available information from their human homologs: LEP (PDB number: 1AX8) and LEPR (PDB number: 3V6O). (**E**) STAT3 reporter assay. 293FT cells were treated with WT and mutant recombinant rat LEP proteins at different concentrations after transient transfection of pcDNA-Lepr, pRL-TK and pGL6-Stat3. Relative luciferase activity was determined by firefly luciferase light units normalized by that of renilla luciferase.

Since these *Lep*^∆*I14/*∆*I14*^ rats display the LEP deficiency phenotypes, we hypothesized that I14 plays an important role in LEP-LEPR signaling pathway. We first examined the transcripts of the mutant *Lep* in *Lep*^∆*I14/*∆*I14*^ rats. Real-time RT-PCR of the total RNA extracted from white adipose tissue (WAT) showed that the mRNA level of *Lep*^∆*I14*^ is 1.6 fold higher than that of the controls. The three-nucleotide deletion on the mRNA of *Lep*^∆*I14*^ was verified by the Sanger sequencing of RT-PCR products (Fig. 5A). In addition, the Western Blot showed that the mature LEP^∆I14^ protein in WAT of the *Lep*^∆*I14*^ rats is significantly higher than that of the controls (Fig. 5B). Elevated expression of *Lep* at both mRNA and protein level results in a higher level of serum LEP in mutant rats than in WT as detected by ELISA (Fig. 5C). Our results indicate that the production and release of mature LEP^∆I14^ is dramatically increased due to the feedback loop^1^.

To examine the molecular defect of LEP^∆I14^ protein, we did computer assimilation of LEP-LEPR interaction with the available structure information of their human homologs. The modeling suggests that LEP^∆I14^ could not stably bind LEPR due to missing of I14 (Fig. 5D). The molecular docking revealed that wild-type LEP (LEP^WT^) docks flat on LEPR mediated by helix A and the C-terminus of LEP^WT^. Two residues on helix A in LEP^WT^ greatly facilitate the binding process, in which K15 interacts with D475 in LEPR whereas Q4 with P526. F41 in A-B loop interacts with D617. At the C-terminus of LEP^WT^, D141 in helix D is surrounded by the pocket of I434, C473, T527, C528 and V529 in LEPR within a contact distance of 4 Å. L142 and S143 contacts N433 and S435 of LEPR mainly by H-bonds. However, LEP^ΔI14^ docks on LEPR with completely opposite orientation with the α-helixes almost vertical to LEPR. In computer modeling, there are three domains in LEP^ΔI14^ interacting with LEPR. On helix A, R20, D23 and I24 mainly contacts N433, S435 and S450 in LEPR by H-bonds. Near the helix B-C loop, R71 and Q75 contacts with N433 and I434 in LEPR. In the helix C-D loop, E115 contacts with R612 in LEPR by salt bridge, where A116, T121 and E122 residues locate near R573, R612, W622 and W625 in LEPR with a distance within 4 Å. Thus, the interface change most likely weakens the stable binding and interaction between LEP and LEPR which are required for the activation of downstream signaling cascades.

As JAK2/STAT3 pathway plays major role in glucose and energy homeostasis, we next tested the effects of LEP^∆I14^ on the STAT3 signaling pathway using luciferase reporter assay. We expressed and purified both WT and LEP^∆I14^ recombinant proteins, then supplemented these two proteins to 293FT cells transiently transfected with the plasmid encoding LEPR and STAT3 luciferase reporter. As shown in Fig. 5E, in contrast to efficient activation of luciferase reporter by LEP^WT^ (EC50 = 3.0 * 10^−9^ M), LEP^∆I14^ treatment failed to activate the STAT3 signaling, presumably due to its inability to activate LEPR. We therefore concluded that deletion of I14 in the mature LEP impairs the LEP-LEPR interaction and the downstream JAK2/STAT3 pathway.

## Discussion

In this study, we succeeded in making a set of *Lep*- and *Lepr*- mutant rats by CRISPR/Cas9 genomic editing technique. When CRISPR/Cas9 was initially used for making transgenic mice, there is a concern about the potential OTE compounding the phenotype of transgenic animals^27^,^31^. To avoid OTE, many groups have developed a variety of strategies when designing the CRISPR/Cas9 sgRNA constructs. First of all, computation programs can help to select optimal sgRNA target sequences in a given gene^32^,^33^,^34^,^35^,^36^ and potential OTS in a given genome^35^,^37^,^38^. OTE can also be reduced either by modification of sgRNA, i.e., addition of guanine nucleotides at the 5′ end^39^ and truncation^40^, or via Cas9 modifications, i.e., paired Cas9 nickase^39^,^41^, and dCas9-FokI^42^,^43^ and direct usage of Cas9 protein^44^. After Cas9-gRNA transfection, OTE is often validated by the conventional PCR based target sequencing^27^,^31^,^39^, although new techniques for genome-wide screening of OTE were developed to facilitate analysis^45^,^46^. Since we used the out-bred SD rats in contrast to the in-bred animals with identical genetic background, genetic variation should be taken into consideration as it was reported that a single SNV could generate OTS in human stem cells^47^. To address OTE concern, we performed whole genome sequencing in two *LEP* mutant founder rats. Using all the available rat genome sequences as well as dbSNP database, we performed systematical analysis of genome sequences and found no OTS in the sampled mutant rats. Our results are consistent with the notion that CRISPR/Cas9, when designed properly, very scarcely introduces OTS in transgenic animals.

In this study*, Lep*^∆*I14/*∆*I14*^ mutant rats displayed similar phenotypes to those *Lep* knockout rats reported previously^24^,^25^, such as high food intake, elevated body weight, and high serum metabolites, e.g., triglyceride (TG) and cholesterol. Interestingly, we also found that *Lep*^∆*I14/*∆*I14*^ rats have late-onset of nephropathy, such as glomeruli hypertrophy, dilated tubules, and deposition of protein casts (Fig. 4B). Since diabetic nephropathy mouse models are restrained in certain strains (e.g., BTBR) and usually required disruption of additional genes (e.g., *eNOS*)^48^, the *Lep*^∆*I14/*∆*I14*^ rats would provide a better and effective model system for studying diabetic nephropathy.

I14 in LEP is highly conserved among different species^49^, however, its critical role in LEP function is previously unknown. Here, we show that I14 is essential for LEP-LEPR interaction and the downstream signaling pathways. In white adipose tissue of *Lep*^∆*I14/*∆*I14*^ rats, *Lep*^∆*I14*^ is highly expressed at both mRNA and protein levels due to the feedback loop. Western blot and ELISA results indicated that high level of mature LEP^∆I14^ protein in *Lep*^∆*I14/*∆*I14*^ rats is continuously processed and secreted into the blood, whereas LEP^WT^ in WT controls is kept in the reservoir of premature protein during fasting (Fig. 5). Our computer assimilation also implicates that I14 is of great importance for the interaction between LEP and LEPR. The 146-aa mature LEP consists of four hydrophobic helices^50^, whereas the LEPRb ectodomain possesses a cytokine receptor homologous domain 1 (CRH1), an Ig-like domain (IGD), a CRH2, and two consecutive F3 domains. In both 2:2 interaction model^51^ and a recent proposed 4:4 complex model^52^, LEP binds primarily to the CRH2 domain on one LEPRb with interaction with IGD on the other neighboring LEPR^51^,^53^. The helices A and C in LEP interact with CRH2 (aka LEP binding domain, LBD) of LEPRb through van der Waals forces facilitated with several hydrogen bonds^49^,^53^,^54^. As modeled in Fig. 5D, ∆I14 disrupts the docking conformation dramatically so that the helices A and C in LEP cannot parallel with CRH2 in LEPRb, which may also affect LEP binding to IGD through its helix D. Altogether, our data suggested that I14 plays an essential role to maintain the secondary structure of Helix A in LEP for its binding affinity to LEPRb.

We found that both adult male and female *Lep* mutant rats generated by CRISPR/Cas9 were infertile, which is a common phenotype reported previously in LEP deficiency animals^2^ and human beings^5^,^12^. LEP is one of the key mediators that bridge nutritional axis with reproductive axis. It is shown that LEP triggers the reproductive signaling cascade through premammilary ventral nucleus (PMV), together with other nitric-oxide releasing sites, i.e. preoptic area (POA), arcuate nucleus (ARC), and dorsomedial hypothalamus (DMH), which stimulates the downstream gonadotropin-releasing hormone (GnRH) and kisspeptin, neurokinin B, and dynorphin (KDNy) neurons in hypothalamus. LEP fulfills its function in hypothalamo-pituitary-gonadal (HPG) axis with GnRH neurons in the center, where agouti-related peptide/neuropeptide Y (AgRP/NPY) and proopiomelanocortin (POMC) neurons project onto KNDy neurons and AgRP/NPY neurons onto GnRH neurons as an alternative pathway^55^. In male mutant rats, the resulting increase in the production of GnRH stimulates the secretion of two hormones, i.e., follicle-stimulating hormone (FSH) and luteinizing hormone (LH), which stimulates Sertoli cells for spermatogenesis and Leydig cells for steroidogenesis, respectively^56^,^57^. The hormonal defects provide an explanation to the histological defects we observed in testes from *Lep* mutant rats (Fig. 4C). *Lep* mutant rats also provide an animal model to study reproduction problems as well.

In summary, we have generated *Lep*- and *Lepr*- mutant rat models by CRISPR/Cas9 to study obesity related diseases. Of particular interests, we showed that I14 in LEP is essential for LEP-LEPR interaction and biological function, and *Lep*^∆*I14/*∆*I14*^ rats recapitulate expected mutant phenotypes of LEP null animals. Future crystal structural study of LEP^∆I14^-LEPR interaction would directly reveal the defective ligand/receptor interaction due to deletion of this 14th Ile in mature LEP.

## Methods

### *In-vitro* transcription of Cas9 mRNA and sgRNAs

Cas9 and sgRNA bicistronic expression vector (pX330 from Addgene) was digested with Bbs I (NEB) and gel purified with MinElute Gel Purification Kit (Qiagen). Each oligo pairs of the targeting site were phosphorylated by T4 Polynucleotide Kinase (T4 PNK, NEB) and annealed by the decline of temperature from 95 °C to 25 °C at 0.1 °C/S. The ligation reaction of the linearized vector and sgRNA sequence was performed with T4 DNA ligase (NEB). All the constructs were verified by Sanger sequencing.

The generation of Cas9 mRNA and sgRNAs was done following the previous protocol^26^. In brief, *in-vitro* transcription of T7-Cas9 PCR product and each T7-sgRNA PCR product was performed by using mMESSAGE mMACHINE T7 ULTRA kit (Life Technologies) and MEGAshortscript T7 kit (Life Technologies), respectively. Cas9 mRNA was purified using MEGAclear kit (Life Technologies), while the sgRNAs were purified by ethanol precipitation. RNA was eluted in RNase-free water (Life Technologies) for intracytoplasmic microinjection in rat zygotes.

### Cell culture and T7 Endonuclease I (T7EI) assay

Rat BRL cells were grown in high glucose DMEM (HyClone) supplemented with 10% FBS (Gibco) at 37 °C with 5% CO2. The CRISPR constructs targeting 2 different sites of *Lep* and *Lepr* were respectively transfected into BRL cells with Lipofectamine 2000 (Life Technologies). T7EI assay was adapted from the previous protocol^58^. Two days post transfection, genomic DNA was extracted from BRL cells. PCR primers (LEP F, LEP R, LEPR1 F, LEPR1 R, LEPR2 F and LEPR2 R) were designed to specifically amplify ~600 bp DNA fragments flanking each targeting site (Detailed primer information is in Supplementary Materials, Table 3). PCR was performed by Q5 High-Fidelity DNA Polymerase (NEB) with the parameters as follows: 98 °C for 30 S; 35 cycles of 98 °C for 10 s, 60 °C for 15 s, 72 °C for 20 s; 72 °C for 2 min. Immediately after PCR, the products were denatured at 95 °C for 5 min, then annealed by temperature decline from 95 °C to 85 °C at 2 °C/s and from 85 °C to 25 °C at 0.1 °C/S. Each re-annealed samples were split into two reactions, of which one contained T7EI (NEB) and the other used water as control. Following incubation at 37 °C for 15 min, the digested PCR products were separated on 12% PAGE gel.

### Animal care and intracytoplasmic RNA microinjection

SD rats were raised in the animal facility at Tongji University. All the protocols were in accordance with the guidelines of Tongji University’s Committee on Animal Care and Use. If not otherwise specified, the rats in static cages were kept in a 12-h light, 12-h dark cycle with *ad libitum* access to food and water. All the experimental procedures as described below were approved by the animal experiment administration committee of Tongji University (# TJLAC-014-014).

Each of SD female rats was hormone-primed by first injection of 40 IU pregnant mare serum gonadotropin (PMSG) and after 48 hours followed by the second injection with 40 IU human chorionic gonadotrophin (hCG). After mating with stud male SD rats overnight, female donors were sacrificed to collect fertilized one-cell-stage zygotes at noontime (0.5 day post coitum, dpc). The mixture of sgRNA and Cas9 mRNA was microinjected into the cytoplasm of one-cell embryo. Shortly after microinjection, the survived embryos were implanted in the oviduct of pseudo-pregnant SD female rats. Full-term pups were obtained by at 21.5 dpc.

### Genotyping

Toe biopsies were collected for genomic DNA extraction and analysis following the approved procedure protocol. PCR primers were the same as those previously described in T7EI assay. PCR was performed by Q5 High-Fidelity DNA Polymerase (NEB) with the parameters as follows: 98 °C for 30 S; 35 cycles of 98 °C for 10 s, 58 °C for 15 s, 72 °C for 20 s; 72 °C for 2 min. The PCR products were then subcloned into the pZeroBack/Blunt vector (Tiangen Biotech). After transformation, 10 positive colonies were picked for Sanger sequencing.

### Whole-genome sequencing

One WT control SD rat, one *Lep* mutant SD rat (#17) and one *Lepr* mutant SD rat (#23) were subjected to whole genome sequencing. Genomic DNA was extracted from the tails of rats. DNA quality was assessed by A260/280 ratio and gel electrophoresis and its quantity was determined by Qubit 2.0 (Life Technologies) and Bioanalyzer 2100 (Agilent). For sequencing library preparation, 1 ug genomic DNA were sheared to fragments of 300–400 bp, end-repaired, A-tailed and ligated to Illumina sequencing adapters. The ligated products of 400–500 bp were size selected on a 2% agarose gel and amplified by LM-PCR. The resulting library was sequenced in 2*100 bp paired-end mode by Illumina Hiseq2000 according to the manufacturer’s recommended protocol. All sequencing reads from this study have been submitted to the European Nucleotide Archive (ENA) under accession number PRJEB7397.

### Reads alignment and variant calling

Quality of raw sequencing reads were checked by NGS QC Toolkit v2.3.1^59^. The reads were trimmed at the end if Phred scaled base quality score dropped below 20. High quality reads were aligned to UCSC rn5 (RGSC Rnor_5.0) using Burrows-Wheeler Aligner (v0.7.5a). BAM files generated from sequencing alignment were preprocessed by Picards toolkits (v1.102) to remove PCR duplicates. GATK software package (v2.7) was used to locally realign reads for SNV and short indel calling^60^. The quality of variant calling was measured by several mapping statistics, including mapping quality score, mapped reads, variant reads, and variant confidence, etc. The low-quality SNV/indels were further filtered out by GATK recommended conditions^60^.

### Off-target site screening and validation

Reference genome was searched for genome-wide Cas9/gRNA target loci by CasOT^38^. We used NGG or NAG as protospacer-adjacent motif (PAM), and chose loci that contain 0–3-base mismatches in the seed region and 0–6-base mismatches in the non-seed region. This low threshold was used to detect as many potential loci as possible. Considering the difference of genetic background between the reference (RGSC Rnor_5.0) and SD rat, we sequenced the WT SD rats and repeated the off-target searching process. Variations in mutant rats were filtered to remove variants in the WT rat, 28 published rat genomes^61^, and 2 previous sequenced rats (unpublished data) and dbSNP database. To reduce errors in indel calling, we filtered out the indels: 1) in repeat regions reported by RepeatMasker or WindowMasker; 2) with 10-bp upstream/downstream sequence as homopolymer; 3) with GC content of 100-bp flanking sequence lower than 20% or higher than 60%. We defined the indels near the gRNA targeting sequence as the potential off-target sites (OTS). These OTS were amplified by PCR with specific primers (Supplementary Materials, Table 3) from genomic DNA of both WT and mutant rats. As described previously in Genotyping section, The PCR products were subcloned and sequenced via Sanger sequencing to further confirm the OTS.

### RT-PCR and Western blot

RT-PCR was performed following the routine protocol. Both RNA and protein from subcutaneous fat pads was extracted by TRIzol (Life Technologies). The first strand cDNA was generated using RevertAid First Strand cDNA Synthesis Kit (Thermo Scientific). PCR primers (LEP RT F and LEP RT R) were designed to specifically amplify 501bp *Lep* cDNA (Supplementary Materials, Table 3). PCR parameter was exactly the same as that in Genotyping section. Real-time RT PCR was performed with *Gapdh* as internal control (Supplementary Materials, Table 3) on the StepOnePlus (Applied Biosystems). Western blot was done following the routine method. The primary antibodies were anti-GAPDH (1:200; XianZhi) and anti-LEP (1:200; Santa Cruz #sc-842), while the secondary antibody was goat anti rabbit (1:1000; Cell Signaling #7074).

### Body weight, food and water consumption

WT, *Lep*- and *Lepr*- mutant rats were given *ad libitum* access to a standard laboratory chow diet and water. The rats were weighted every other week during the time course. Average daily intake was calculated by weighting the food at certain week during the time course.

### Serum chemistry, glucose tolerance test, and serum INSULIN and LEPTIN levels

The rats were fasted for 16 h, and blood was collected by tail vein puncture and the serum chemistry parameters were analyzed at Shanghai Changzheng Hospital, the Secondary Military Medical University.

For glucose tolerance test, the animals were fasted for 6 h before administered ip injection with d-glucose at 2 g/kg, and blood was collected by tail vein puncture. The glucose levels were measured by Accu-Chek Performa (Roche).

The animals were fasted for 16 h and blood was collected by tail vein puncture. Blood was left to clot for 1 h at room temperature, and serum was obtained after centrifugation. The serum INSULIN levels were measured by Rat/Mouse Insulin ELISA Kit 96-well plate (Millipore). The serum LEPTIN level was measured by Rat Leptin ELISA Kit (Sigma).

### Subcutaneous infusion of recombinant LEP

Each *Lep*^∆*I14/*∆*I14*^ rat was subcutaneously implanted an Alzet osmotic minipump (model 2001, Durect) filled with rat recombinant LEP (Genscript) at a dosage of 200 μg/kg/day (in sterile saline), while each WT rat was subcutaneously implanted an Alzet osmotic minipump (model 2001, Durect) filled with sterile saline as control. After 7 days, the minipumps were removed from both WT and *Lep*^∆*I14/*∆*I14*^ rats except that an Alzet osmotic minipump (model 2001, Durect) filled with sterile saline was subcutaneously implanted into each *Lep*^∆*I14/*∆*I14*^ rat afterwards. The subcutaneous implantation site was chosen on the back of animals, between and slightly posterior to the scapulae. All the procedures were performed by following the manufacturer’s (Durect) protocol. The body weight and food intake were measured as described above.

### Molecule docking

The structures of LEP (PDB number: 1AX8) and LEPR (PDB number: 3V6O) were downloaded from the RCSB (Research Collaboratory for Structure Bioinformatics). The structure of LEP^∆I14^ was modeled using LEP as the template through the Swiss-PDB-viewer software. The candidate complex structure was generated from the previously modeled structures by the ZDOCK program in Discovery Studio 2.5. Through clustering analysis, the most possible hit was selected for further study.

### STAT3 reporter assay

293FT cells were cultured in DMEM as described above. The cells were seeded in a 96-well plate (Corning) and transfected with the human LEPR overexpression vector pcDNA-Lepr, the control pRL-TK (Promega) and the pGL6-Stat3 plasmid which contains the luciferase gene under control of the STAT3-inducible promoter. One day after transfection, the cells were treated with different concentrations of WT and mutant recombinant rat LEP proteins (encoded by cDNA inserted between BamH I and Sal I sites of pET-21a(+)), which were produced following the previous report^62^. LEP-induced luciferase activity was measured by Dual-Glo Luciferase Assay System (Promega) following the manufacturer’s manual. Luciferase activity was measured in GloMax-Multi+ (Promega). Statistical analysis was done using Prism (Graphpad Software).

## Additional Information

**How to cite this article**: Xu, S. *et al*. The 14th Ile residue is essential for Leptin function in regulating energy homeostasis in rat. *Sci. Rep.* **6**, 28508; doi: 10.1038/srep28508 (2016).

## Supplementary Material

Supplementary Information

Supplementary Table S1

## Figures and Tables

**Table 1:** Generation of knockout rats via the CRISPR/Cas system.

Construct	Embryos transferred	Newborns	Mutant		Homozygote	Compound heterozygote	Mosaic
			Male	Female			
Lep1	26	23	5	7	6	6	0
Lep1*	75	19	9	10	0	1	7
Lep2	20	5	4	1	1	4	0
Lepr1	40	8	2	0	1	1	0
Lepr2	40	9	0	0	0	0	0

^*^The injection of Lep1 gRNA and Cas9 mRNA with a lower concentration of 25 ng/μL each, compared to the other injections whose gRNAs and Cas9 mRNA were at 50 ng/μL and 100 ng/μL respectively.

**Table 2:** Fasting serum chemistry of *Lep*^*ΔI14/ΔI14*^ rats at 16 wk of age.

		Triglycerides (mmol/L)	Cholesterol (mmol/L)	HDL (mmol/L)	LDL (mmol/L)
Male	WT	1.17 ± 0.39	0.49 ± 0.15	0.38 ± 0.13	0.30 ± 0.11
	*Lep*^*ΔI14/ΔI14*^	1.94 ± 0.56*	3.40 ± 2.02* *	0.54 ± 0.12	0.33 ± 0.14
Female	WT	1.27 ± 0.35	0.42 ± 0.15	0.43 ± 0.10	0.27 ± 0.11
	*Lep*^*ΔI14/ΔI14*^	2.86 ± 0.78* *	4.93 ± 1.65* * *	0.58 ± 0.11*	0.38 ± 0.08

HDL, High-density lipoprotein; LDL, low-density lipoprotein.

Values are means ± SD. (Male: n = 6; Female: n = 6 for WT and n = 5 for Mut).

*P < 0.05 vs. controls. **P < 0.01 vs. controls. ***P < 0.001 vs. controls.
