# Supplementary material for: The 14th Ile residue is essential for Leptin function in regulating energy homeostasis in rat
Source: Sci Rep. 2016 Jul 5;6:28508. doi: 10.1038/srep28508 (PMC4932527; doi:10.1038/srep28508)
Supplement: Supplementary Information [file srep28508-s1.pdf]

Supplementary information

The 14th Ile residue is essential for Leptin function in regulating energy homeostasis in rat

Shuyang Xu[1,†], Xianmin Zhu[1,†], Hong Li[2,†], Youtian Hu[3], Jinping Zhou[4], Di He[1], Yun Feng[4], Lina Lu[4], Guizhen Du[5], Youjin Hu[4, 5], Tiancheng Liu[2], Zhen Wang[2], Guohui Ding[2], Jiayu Chen[1], Shaorong Gao[1], Fang Wu[3], Zhigang Xue[4], Yixue Li[2], Guoping Fan[1,5,*]

*To whom correspondence should be addressed at: Tongji University, School of Life Sciences and Technology, 1239 Siping Road, Shanghai 200092, China

TEL: +86 21 65982417; Email: guopingfan@gmail.com

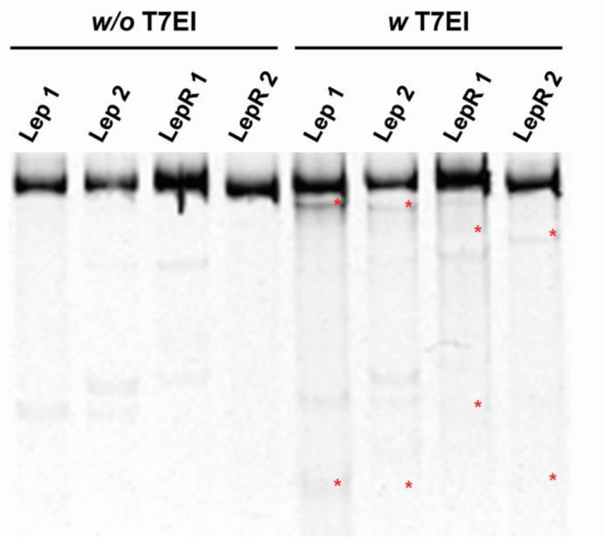

Supplementary Figure S1: The T7 endonuclease I (T7EI) assay.

Lep 1, Lep 2, LepR 1 and LepR 2 CRISPR constructs resulted in target-specific mutation after in-vitro transfection of BRL cells. Compared to the controls (w/o T7EI), PCR products were cleaved by T7EI with expected fragment sizes (indicated by red asterisks *)

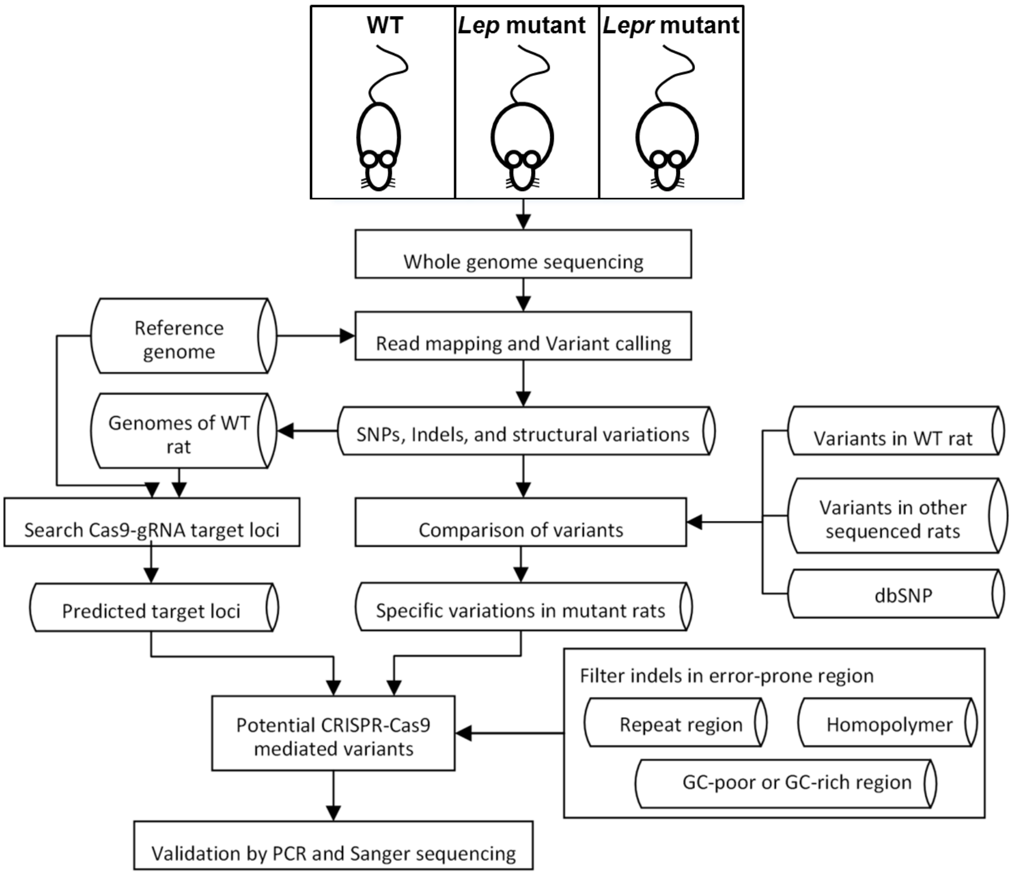

Supplementary Figure S2: Pipeline for whole-genome sequencing and computational analysis of CRISPR-Cas9 off-target effect.

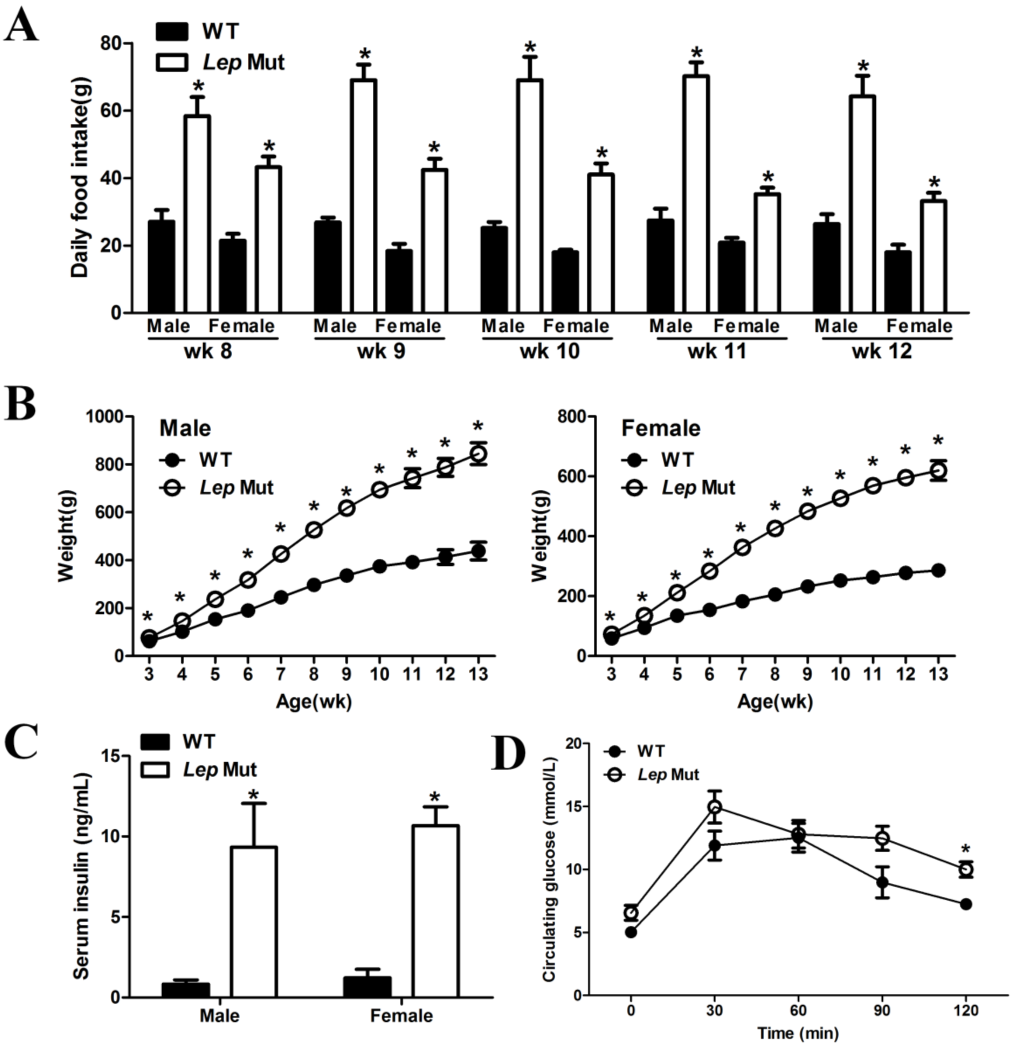

Supplementary Figure S3: LEP-deficiency induced obesity, glucose intolerance, and hyperinsulinemia in the founders.

(A) Daily food intake (Means ±SD) for WT (n=5) and *Lep* Mut (n=5) measured over 5 weeks. *, *P* < 0.05 *vs*. controls.

(B) Body weight was measured over 13 weeks for WT (n=5) and *Lep* mutant (n=9) males, and WT (n=6) and *Lep* mutant (n=8) females. *, *P*< 0.01 *vs*. controls.

(C) Serum insulin levels in 40-wk-old WT and *Lep* Mut rats (n=5 for each group). *, *P* <0.05 *vs*. controls.

(D) Male WT and *Lep* mutant rats (n=5 for each) at age of wk-10 were ip injected with D-glucose, and serum glucose levels were determined at 0, 30, 60, 90, and 120 min after administration. *, *P*< 0.05 *vs*. controls.

Supplementary Table 2 Fasting serum chemistry of *Lep* mutant founders at 16 wk of age

		Triglycerides (mmol/L)	Cholesterol (mmol/L)	HDL (mmol/L)	LDL (mmol/L)	Glucose (mmol/L)
Male	WT	1.08±0.46	1.88±0.32	0.68±0.06	1.54±0.05	3.58±0.21
	Lep Mut	2.45±1.35*	5.36±1.28*	1.85±0.32*	2.06±0.32*	4.27±0.62*
Female	WT	0.38±0.03	2.3±0.18	0.83±0.03	1.62±0.03	3.83±0.31
	Lep Mut	4.34±1.08*	3.36±0.58*	1.01±0.24	1.63±0.13	5.00±1.61

HDL, High-density lipoprotein; LDL, low-density lipoprotein.

Values are means±SD. (Male: n=4 for WT and n=7 for Mut; Female: n=3 for WT and n=7 for Mut)

* $P < 0.05$ vs. controls.

Supplementary Table 3 Primers for PCR amplification.

Primer	Sequence	Product Size(bp)
Primers for sgRNA target regions, *Lep* RT-PCR and qPCR		
Lep F	AGCGAGGAAAATGTGCTGGA	600
Lep R	AGGAAGGGGAGAGAGTGGTC	
Lepr1 F	CACACTCGTACACAGGCATG	591
Lepr1 R	TGGAGCCTTAATGCAGAGCT	
Lepr2 F	GGAGGAGAGATGCCAGGGAA	520
Lepr2 R	CAGCGTCTTCCCTTCAGTGT	
Lep RT F	ATGTGCTGGAGACCCCTGT	501
Lep RT R	TCAGCATTCAGGGCTAAGGT	
GAPDH qPCR F	GGGAAGCTGGTCATCAACGG	110
GAPDH qPCR R	ACGCCAGTAGACTCCACGAC	
Lep qPCR F	TCCAGGATGACACCAAAACCCT	123
Lep qPCR R	GGTGAAGCCCGGGAATGAAG	

Primers for WG off-target regions		
17-1F	AGGATCAATGAAAACGCCCC	505
17-1R	AGAGTCAGAGTCCTCCCGTA	
17-2F	GTAACAGAAATGGCCGCGAT	567
17-2R	TCAAGTGTTTTATGGCTGAGCT	
17-3F	CCAGGCCCAGCACTATTCTT	550
17-3R	AGCCTCTCTCTGCACCAAAT	

23-1F	GAGGGAAATCACAGGGGTCA	530
23-1R	CATTTGCCCTCCATCTCTGC	
23-2F	AATCCACCATCAGCCAGGAA	549
23-2R	TCAGGTTGTGTCAAAGCAGC	
23-3F	GGACCACGTTGTAACAGCTT	595
23-3R	GGAGGTGTAGACATGTCCCC	
23-4F	GCGAGTATCTCTTTGAGCGC	507
23-4R	AGCCTAATCTGTGGTCCTTCC	
23-5F	TCCCCTAATCTTCCTGCCTT	591
23-5R	AGCCATCAACCAGGAGCATA	
23-6F	CCCCACACCTACCTTAACAGT	537
23-6R	GGCAAGATGTCACCTCTCCT	
23-7F	CTGAACTGTGTGGTGCTGG	502
23-7R	CACAGGGCCAACATAGACCT	

Primers for Lep1 gRNA off-target regions

Lep1-OT1-F	CAATGCACCACTGTCACACA	650
Lep1-OT1-R	CGACGGCTCTAAGGACAGAA	
Lep1-OT2-F	CTAGGTAAAGCGATGCAGGC	665
Lep1-OT2-R	CCCTTCTCCTCAGCTGTCTC	
Lep1-OT3-F	CTCACGAGTGGCTTTGTTCC	645
Lep1-OT3-R	ACCCAAGCTTCTCTACCCAC	
Lep1-OT4-F	CCATGCTTCTCACCACGATG	692
Lep1-OT4-R	TGCACACCAGAACCCTATGT	
Lep1-OT5-F	TTGTGTGAGGTAGTGGCTGT	582
Lep1-OT5-R	AGCAGTCTTCATGCTTGGG	
